# Role of the Interventional Radiologist in the Treatment of Desmoid Tumors

**DOI:** 10.3390/life13030645

**Published:** 2023-02-26

**Authors:** Daniel Goldberg, Gregory Woodhead, Jack Hannallah, Shamar Young

**Affiliations:** Department of Medical Imaging, Division of Interventional Radiology, University of Arizona Medical Center, Tucson, AZ 85712, USA

**Keywords:** desmoid tumor, cryoablation, ablation, transarterial chemoembolization, radiofrequency ablation, microwave ablation, HIFU

## Abstract

Desmoid tumors are locally aggressive soft tissue tumors with variable clinical presentation. As is the case with most relatively rare tumors, a multidisciplinary team approach is required to best manage these patients. Surgical resection, systemic therapy, and radiation therapy have classically been mainstays of treatment for desmoid tumors; however, a more conservative “wait-and-see” approach has been adopted given their high recurrence rates and significant morbidity associated with the aforementioned therapies. Given the challenges of classical treatment methods, interventional radiologists have begun to play a significant role in minimally invasive interventions for desmoid tumors. Herein, the authors review imaging characteristics of desmoid tumors, current management recommendations, and minimally invasive therapeutic intervention options.

## 1. Introduction

Desmoid tumors, also called aggressive fibromatosis, are rare, locally invasive tumors with no potential for metastasis. However, they are locally aggressive, demonstrating proliferation of myofibroblasts, and can occur anywhere in the body. There is an estimated annual incidence of 2–4 new cases per million people and they most frequently occur between the ages of 15 and 60 years, with a peak incidence in the third and fourth decade of life [1]. The classification of desmoid tumors is based on their location. This leads to two types of desmoid tumors, namely extra and intra-abdominal desmoid tumors, which are typically separated given the difference in their disease course.

Extra abdominal desmoid tumors typically present as slow-growing soft tissue masses that may be painful. As these masses enlarge, they may compress neurovascular structures, which often serves as a driving factor in their symptomatology. Intra-abdominal desmoid tumors are also typically slow growing and may present with pain. However, given their anatomic location adjacent to critical structures such as the bowels, they are associated with bowel ischemia and obstruction secondary to tumor enlargement and compression.

Desmoid tumors can arise sporadically or can occur in association with familial adenomatous polyposis, specifically Gardener’s Syndrome [2]. The majority (approximately 90%) of desmoid tumors occur sporadically in extra-abdominal locations such as the head, neck, thorax, and extremities. Factors such as surgery, pregnancy, trauma, and oral contraceptive use have shown an association with sporadic desmoid tumors [3]. The sporadic form of desmoid tumor presents with mutations in the *CTNNB1* gene, part of the Wnt signaling pathway [4]. The *CTNNB1* gene mutation can be utilized to differentiate desmoid tumors from benign fibroblastic and myofibroblastic lesions as well as low grade sarcomas [5]. Unlike sporadic desmoid tumors, familial adenomatous polyposis associated desmoid tumors result from mutations in both the wild-type *CTNNB1* and adenomatous polyposis coli (APC) genes [4]. Individuals with familial adenomatous polyposis are a thousand-fold more likely to develop a desmoid tumor compared with the general population [3]. Additionally, multifocal disease is not uncommonly seen in patients with familial adenomatous polyposis, while being very rare in sporadic cases [2].

Classically, desmoid tumors were managed aggressively with medical, surgical, and radiation therapy. This treatment method has largely been reworked, as it unfortunately resulted in suboptimal outcomes. A recent shift in the management paradigm to a less aggressive “wait and see” approach has occurred; with this shift minimally invasive therapeutic interventions have also increased in prevalence in symptomatic desmoid tumors. Previously explored minimally invasive techniques include multiple different ablation modalities as well as transcatheter embolization. This article will review relevant imaging features of desmoid tumors, elucidate the modern management approach to desmoid tumors, and discuss current minimally invasive therapeutic interventions being explored to treat desmoid tumors.

## 2. Imaging Characteristics

**Figure 1:**
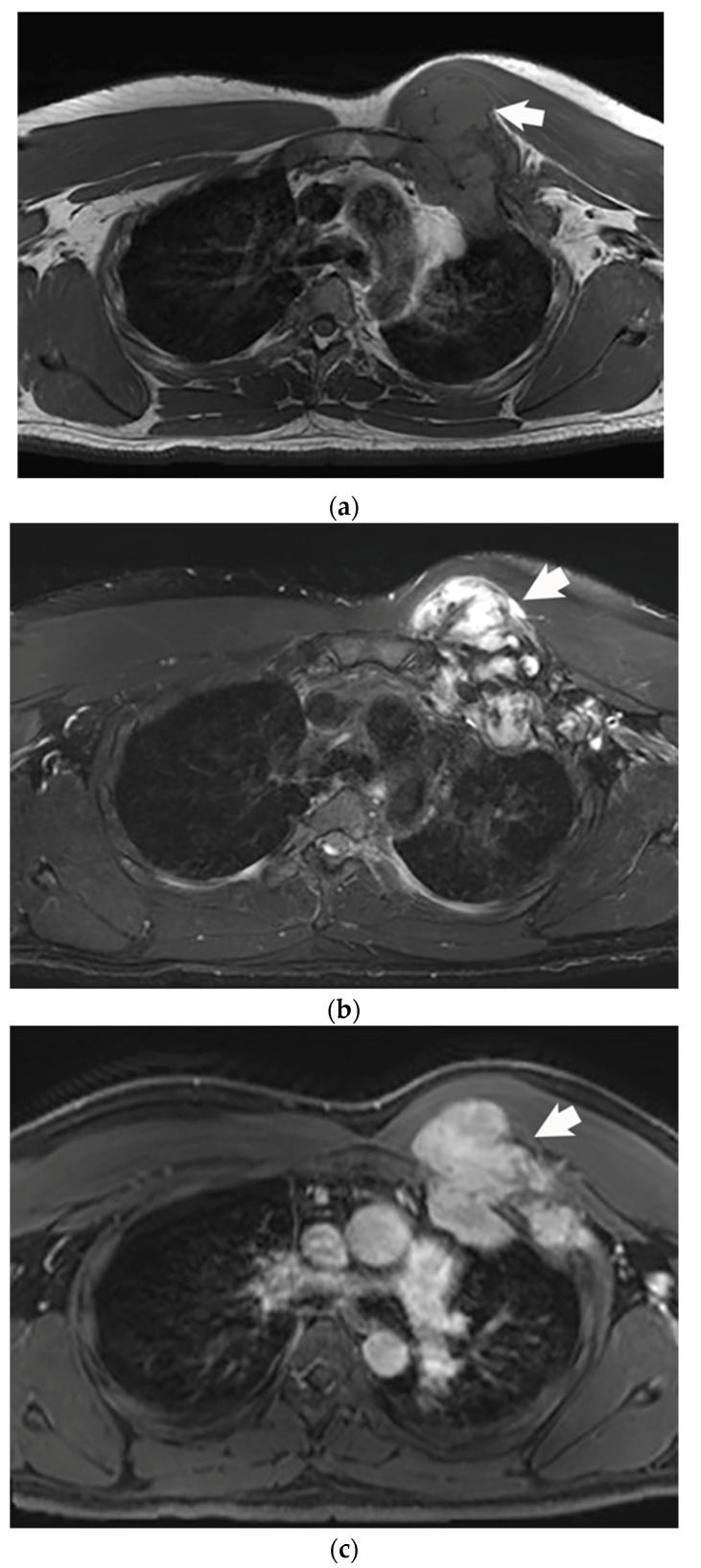
45 year old male with a spontaneous, extra-abdominal desmoid tumor (arrow) involving the left chest wall. (**a**) A selected pre contrast T1 magnetic resonance imaging image demonstrating iso to hypo intensity of the desmoid tumor as compared to the skeletal muscle. (**b**) Selected magnetic resonance imaging image demonstrating predominantly hyperintensity of the desmoid tumor (arrow) on a fat saturated T2 sequence. (**c**) Selected magnetic resonance image demonstrating avid enhancement of the desmoid tumor (arrow) after administration of gadolinium based intravenous contrast.

Ultrasound, computed tomography, and magnetic resonance imaging are the most common imaging modalities used by interventional radiologists for pre-procedural planning, intra-procedurally, and to evaluate post-procedural outcomes (Table 1). On ultrasounds, desmoid tumors present with variable margins ranging from well-defined and smooth to poorly defined or “infiltrative”. Desmoid tumors also demonstrate variable internal echogenicity and vascularity. On computed tomography and magnetic resonance imaging, desmoid tumors may present as well or ill defined (Figure 1). Typical imaging features seen on magnetic resonance imaging included iso to hypointense T2 signal and isointense T1 signal when compared to skeletal muscle [6]. High T2 signal intensity typically implies a high content of spindle cells, while low T2 signal typically represents dense collagen and hypocellularity [7]. Following the administration of intravenous gadolinium-based contrast, desmoid tumors predominantly demonstrate moderate to marked enhancement [8]. Magnetic resonance imaging is the preferred modality for pre procedural treatment planning and post procedural follow-up given its superior soft tissue contrast resolution as compared with computed tomography or ultrasound [6]. Computed tomography and ultrasound are generally preferred for intra-procedural image guidance, given their prevalence and ready availability in most interventional radiology practices.

## 3. Management of Desmoid Tumors

A multidisciplinary approach is recommended for the management of desmoid tumors. Ideally the multidisciplinary team includes a medical oncologist, a radiation oncologist and a surgical oncologist as well as diagnostic and interventional radiologists. The current National Comprehensive Cancer Network guidelines recommend observation with imaging in asymptomatic patients who have a tumor in an anatomic location where progression would not be likely to result in morbidity, and in select patients where progression would be likely to result in significant morbidity [9]. This strategy is proposed in part because the spontaneous regression of desmoid tumors has been observed in as many as twenty percent of cases [10]. Therapy is recommended for symptomatic patients, in patients with tumors in anatomic locations where progression would result in significant morbidity, and in patients where significant disease progression occurs while under a watchful waiting protocol. For intra-abdominal and retroperitoneal tumor locations, systemic therapy and/or surgery are recommended. For extra-abdominal disease, surgery, systemic therapy, ablation, and radiation therapy are all considered appropriate therapeutic options to be used as first or second line therapy, depending on the extent and location of the disease as well as institutional capabilities.

Classically, surgical resection had been the mainstay of desmoid tumor therapy; however, it can be highly morbid and has a 30–50% recurrence rate even when negative margins are achieved [11]. Surgery-related morbidity due to large areas of soft tissue resection to achieve negative margins may result in increased surgical complications, the need for complex surgical reconstruction, and decreased quality of life [12]. Improved local control rates are seen with external beam radiation alone (78%) and adjuvant radiation following surgery (94%) [11]. Although radiation improves local control rates, radiation-related late complications are seen in as many as twenty percent of patients and include fibrosis, fracture, and secondary malignancy [11].

A number of different systemic therapy classes and specific agents, such as hormonal agents (tamoxifen, toremifiene, aromatase inhibitors), anti-inflammatory drugs (sulindac, celecoxib), cytotoxic agents (doxorubicin, vinorelbine, vinblastine, methotrexate), and molecular targeted therapies (sorafenib, pazopanib, imatinib, nirogascestat) have been utilized [13,14]. According to current clinical guidelines, systemic therapy is indicated in cases of rapid tumor growth and when surgical resection is contraindicated. The role of hormonal and anti-inflammatory agents remains controversial. Cytotoxic agent therapy is associated with systemic toxicity that must be balanced against overall quality of life [15]. Furthermore, most systemic regimens have only been assessed in phase II non-randomized trials or retrospective studies, and no supporting evidence-based data elucidating which is the best sequence or combination for available systemic therapies exists [16]. Future trials are unlikely to occur in the future, given the rare and heterogeneous nature of desmoid tumors.

## 4. Ablation

Image guided, percutaneous chemical and thermal ablation techniques have been used to successfully treat desmoid tumors through minimally invasive means. Commonly, ablation has been used as an alternative to surgical resection, given the significant morbidity and high rate of disease recurrence associated with surgical resection [11]. Percutaneous ablation has been used to treat intra-abdominal, abdominal wall, and extra-abdominal desmoid tumors. Furthermore, ablation has been reported in both first line therapy and for disease recurrence after prior therapy. This section reviews both chemical and thermal ablation techniques that have been reported. 

### 4.1. Chemical

The earliest documented image guided, percutaneous ablation of a desmoid tumor involved the use of percutaneous chemical ablation [17]. Chemical ablation is performed by percutaneously injecting chemical agents into tumors using image guidance. Two agents commonly used for the chemical ablation of solid tumors are ethanol and acetic acid. Ethanol is known to cause cytoplasmic dehydration, denaturation of cellular proteins, and microvascular thrombosis, ultimately resulting in the coagulative necrosis of tumor tissue [18]. Ethanol has been used in a number of different ablation scenarios and is known to be very effective at inducing cellular death. However, as with all forms of chemical ablation, ensuring complete coverage of the desired ablation zone is difficult. These difficulties increase as the size of the target lesion increases. Acetic acid is able to infiltrate the septa and capsule of tumor resulting in protein denaturation, dissolution of basement membrane and interstitial collagen, similarly causing coagulative necrosis [19]. Acetic acid suffers from the same concerns as ethanol in terms of ensuring complete coverage of the target area. 

Limited experience with chemical ablation in the treatment of desmoid tumors is available within the literature. Clark et al. described performing chemical ablation of two patients with desmoid tumors using acetic acid with computed tomography guidance: one patient with a recurrent extra-0abdominal desmoid tumor within the mediastinum, and the other patient with an inoperable intra-abdominal tumor within the mesentery [17]. Both patients demonstrated a reduction in tumor size following multiple ablation sessions without post procedural complication. Although a significant reduction in tumor size was achieved, this ablation modality is inefficient, requiring five to six ablation sessions to obtain the reported results.

### 4.2. Radiofrequency

Radiofrequency ablation functions by inducing a localized, alternating electric field in close proximity to the radiofrequency electrode. The alternating electric field produces marked agitation of the tissue ions, resulting in the production of frictional heat around the electrode. This frictional heat induces thermal injury and tissue coagulation [20]. The known limitations of radiofrequency ablation include increased tissue impedance secondary to tissue desiccation and charring resulting in decreased local tissue temperatures as well as less predictable ablation zones in areas of perfusion and ventilation secondary to conductive heat transfer [21]. Radiofrequency ablation also requires a slow increase in delivery of energy, which introduces the possibility of user error. 

The first reported use of radiofrequency ablation to treat a desmoid tumor was by Tsz-Kan et al. who in 2002 performed an ablation on a recurrent extra abdominal desmoid tumor of the back in a patient that refused repeat surgery [22]. Post procedurally, there was no recurrence of the tumor, however, the post-procedural course was complicated by a wound infection. Multiple other case reports and case series have demonstrated encouraging results for using radiofrequency ablation to treat abdominal wall and extra-abdominal desmoid tumors [23,24,25]. Of note, skin burns were observed in several patients, with one requiring skin grafting. Furthermore, Wang et al. described the successful application of radiofrequency ablation in the treatment of a large intra-abdominal desmoid tumor causing bowel compression [26]. This is of interest as intra-abdominal tumors, particularly mesenteric desmoid tumors, are often felt to be poor ablation candidates. 

### 4.3. Cryoablation

**Figure 2:**
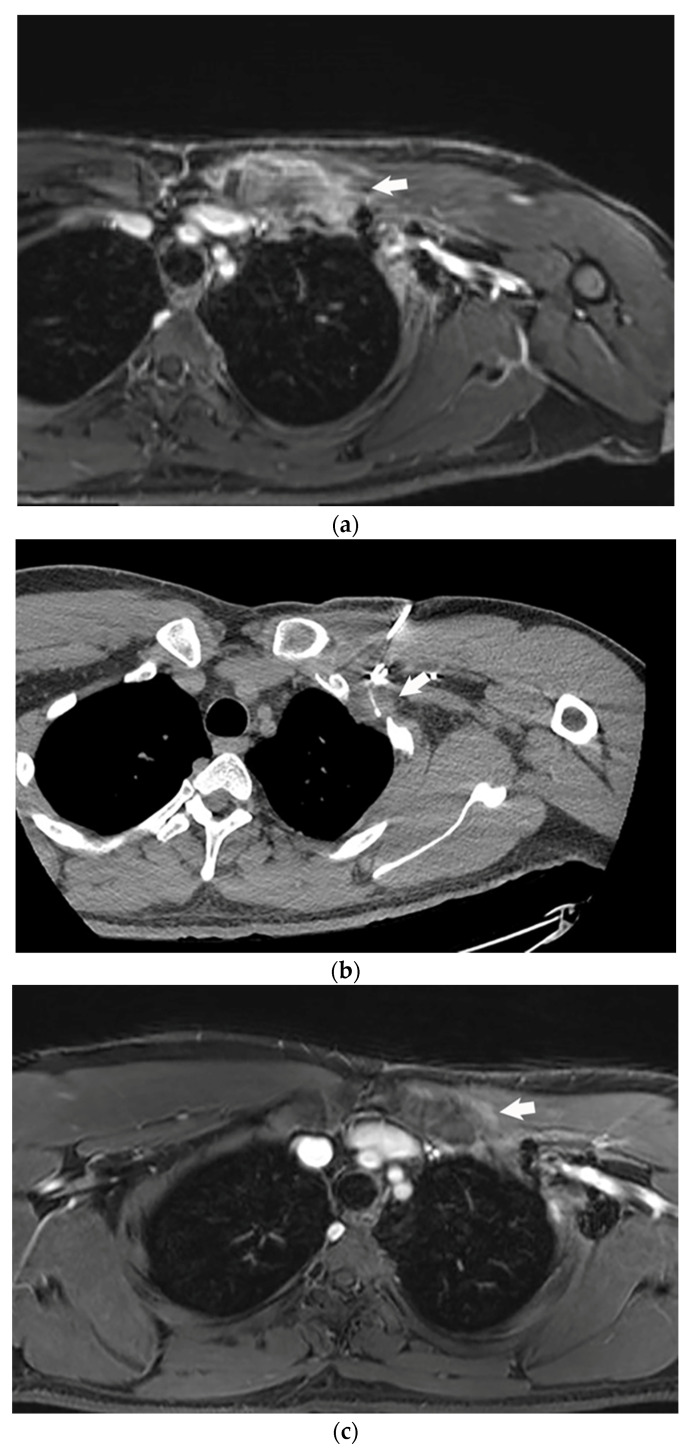
Desmoid tumor patient following surgical resection and cryoablation of residual desmoid tumor. (**a**) Selected post contrast magnetic resonance imaging image demonstrating avid enhancement of residual desmoid tumor (arrow) following surgical resection. The patient also continued to have left upper extremity pain, which was significantly limiting his quality of life. (**b**) Intra procedural computed tomography during percutaneous cryoablation of residual desmoid tumor (arrow) following prior resection. Hypodense ice ball, which roughly approximates the anticipated ablation zone, is seen surrounding cryoablation probe (arrow), within the desmoid tumor. (**c**) Post contrast magnetic resonance imaging demonstrating no evidence of residual desmoid tumor following cryoablation (arrow).

To date, image-guided percutaneous cryoablation of desmoid tumors has been the most widely published ablation modality in the literature. The freezing of tissue causes both extra and intra cellular ice formation, resulting in cell death via direct cell membrane damage, osmotic dehydration, and membrane rupture, as well as vascular damage and thrombosis [27]. Additionally, of the available ablation modalities, only cryoablation offers the benefit of direct intra procedural visualization of the ablation ice ball, helping to better predict the zone of ablation (Figure 2). This provides significant benefit to the performing interventional radiologist in their ability to not only ensure that the entirety of the desmoid tumor is covered, but also that adjacent structures are not included in the ablation zone. 

The first reported use of image guided, percutaneous cryoablation in the literature was by Kujak et al., who performed ablation on five patients with extra abdominal desmoid tumors demonstrating local control in four out of five (80%) patients with small and moderately sized tumors [28]. Since this initial report, several additional groups have presented single institution, cohort studies on their experiences with cryoablation of extra-abdominal desmoid tumors, where between ten and thirty four patients were treated with cryoablation. In this patient cohort, between seventeen and forty four ablations were performed [29,30,31,32,33,34,35,36]. All groups reported that cryoablation was a safe and effective means of local tumor control with several reporting improved post procedural pain control.

Two recent systematic reviews and meta-analyses evaluated cryoablation in the treatment of desmoid tumors. Vora et al. evaluated nine full text papers that included two hundred and fourteen patients with two hundred and thirty four extra abdominal desmoid tumors that underwent two hundred and eighty two cryoablation procedures [37]. They specifically evaluated safety, efficacy, and symptom relief with cryoablation. No deaths were identified. They noted a minor complication rate of 4.8–23.3% and a major complication rate of 2.4–14.2%. The most common major complication encountered was nerve injury followed by rhabdomyolysis, skin necrosis, bleeding, infection, and colo-cutaneous fistula. The most common minor complications included pain, swelling, hematoma, frostbite, and sensory deficit. The estimated one and three year progression free survival was 84.5% (95% CI: 74.6–95.8%) and 78% (95% CI: 63.8–95.3%), respectively. In terms of pain control, pooled analysis demonstrated a visual analogue scale improvement of greater than three in 87.5% (95% CI: 63.8–95.3%) based on two studies’ data. The papers reported partial or complete symptom relief ranged from 37.5% to 96.9%. Overall, cryoablation was found to be safe and effective modality to treat extra-abdominal desmoid tumors. 

A more recent systematic review by Cazzato et al. evaluated five studies that included a total of one hundred and forty six patients looking at the clinical outcomes of cryoablation therapy for extra abdominal desmoid tumors [16]. The mean volume of desmoid tumors ablated was two hundred thirty seven cm^3^. Major and minor complications were seen in 2.4–30% and 7.3–84% of patients across all studies, respectively. All patients were evaluated following cryoablation using the modified response evaluation criteria in solid tumors criteria. Complete response ranged between 0–43.3% and the partial response rate was 26.2–73.9%. Large tumor size was attributed to the low complete response percentage. Local progression free survival at one and three years ranged from 85.1–85.8% and 77.3–82.9% respectively. Complete pain relief was reported in 40–66.7% of patients. The researchers concluded that cryoablation was a safe and effective means of treating extra-abdominal desmoid tumors.

Only one prospective study to date has evaluated cryoablation in the treatment of extra-abdominal desmoid tumors. The CRYODESMO-01 was a prospective, open label, non-randomized, multicenter study that assessed cryoablation in patients with extra-abdominal desmoid tumors progressing under medical therapy [38]. The primary study end point was the non-progression rate at 12 months. Secondary end points included non-progression rates at six and twelve months according to modified response evaluation criteria in solid tumors and magnetic resonance imaging contrast product uptake, safety, functional status, and pain. Fifty patients with extra abdominal desmoid tumors were included in the study and had a mean tumor volume of two hundred and nine cm^3^. The rate of non-progression was 86% (95% CI: 73–94%) at 12 months and the median progression free survival was not achieved at 31 months of follow up. Grade 1, 2 and 3–4 toxicities occurred in 32.8%, 44.5%, and 22% of patients, respectively, with the most common side effects being pain, nerve changes, edema, musculoskeletal impairment, and skin burns. Pain and quality of life scores also improved significantly following cryoablation. 

### 4.4. Microwave

Microwave ablation is another thermal ablation modality that has been used in the treatment of extra abdominal desmoid tumors. Microwave ablation induces tumor cell death in water containing structures through rapid molecular rotation, which in turn generates and uniformly distributes heat throughout the tumor causing thermal injury and coagulation, similar to that of radiofrequency ablation [39]. In contrast to electric currents produced during radiofrequency ablation, microwave ablation can radiate through all biological tissues, irrespective of their conductivity or impedance, allowing continuous heat generation at the antenna resulting in faster, hotter, and larger ablation zones [40]. Microwave ablation also automizes the heating, removing the possibility of user error which can occur in radiofrequency ablation. 

Of the available thermal ablation modalities, the least amount of experience has been reported with microwave ablation. Martinez-Martinez et al. performed percutaneous microwave ablation in nine patients with extra abdominal desmoid tumors involving the thigh, leg, and periscapular region [41]. The mean lesion volume treated was two hundred and twelve point seven cm^3^ and they demonstrated an average tumor volume reduction of 70.4% (SD 24.9%), and an 88.9% improvement in quality of life. Of note, three patients required multiple ablation sessions for further tumor volume reduction and for tumor recurrence. Complications rates were low, with one patient having a post procedural hematoma and one patient suffering a nerve injury. Microwave ablation does appear to be a safe or effective means of treating desmoid tumors; however, experience is limited and more trials are needed.

### 4.5. High Intensity Focused Ultrasound

High intensity focused ultrasound is a noninvasive, heat-based ablation technique that utilizes focused ultrasound beams to heat tissue and produce coagulative necrosis [42]. The benefits of high intensity focused ultrasound generally include its noninvasive nature, lack of ionizing radiation, low morbidity, and good safety profile allowing for multiple ablation sessions if necessary. High intensity focused ultrasound has been performed using ultrasound or magnetic resonance imaging guidance. High intensity focused ultrasound performed using ultrasound guidance provides real time ultrasonographic ablation changes within tissues as well as immediate post ablation assessment of viable tumor when combined with ultrasound contrast. High intensity focused ultrasound performed with magnetic resonance imaging guidance provides the added benefit of tissue temperature monitoring via magnetic resonance thermometry in the near and far treatment fields [42]. As with contrast enhanced ultrasound, guided High intensity focused ultrasound, immediate post contrast imaging can be performed using magnetic resonance imaging to assess for persistent enhancing tumor tissue following ablation.

Ultrasound guided High intensity focused ultrasound ablation was first used in the treatment of ten patients with extra abdominal desmoid tumors by Wang et al. in 2011 [43]. Significant coagulative necrosis was observed in all patients with greater than fifty percent reduction of tumor volume and no major complications. Since then, multiple additional case series have been published with similar results for both extra and intra-abdominal desmoid tumors treated with ultrasound guided High intensity focused ultrasound [44,45,46].

More recently, magnetic resonance guided High intensity focused ultrasound ablation of desmoid tumors has been described in the literature with positive results. Avedian et al. first described using magnetic resonance guided High intensity focused ultrasound to ablate extra abdominal desmoid tumors in nine patients [47]. They reported a thirty five percent decrease in tumor size post ablation, as well as minor complications that included first degree skin burns and localized pain. Multiple additional single institution case series have been published with similar results when treating extra abdominal desmoid tumors [48,49]. However, the largest multicenter cohort study to date involved fifteen patients with extra abdominal desmoid tumors [50]. Changes in viable and total tumor volume were measured following treatment along with adverse events. Following ablation, the median viable targeted tumor volume decreased sixty three percent, while maximum pain scores decreased by sixty four percent post ablation. The most common adverse event was minor skin burns that were attributed to superficial tumor location and prior surgical scar within the treatment regions.

## 5. Transcatheter Embolization

Recently, transarterial chemoembolization has emerged as a promising therapeutic alternative in the treatment of desmoid tumors. The principle of transarterial chemoembolization is based on providing concentrated doses of chemotherapeutic agents to arteries directly supplying a tumor, thereby decreasing possible toxic side effects that may be observed with systemic administration. Transarterial chemoembolization may also be combined with embolization to increase the dwell time of chemotherapeutic agents within tumor beds. This has the added benefit of reducing the concentration of chemotherapeutic seen within the systemic circulation at any given time point. 

Two recent case series have reported successful results using drug eluting bead transarterial chemoembolization loaded with doxorubicin for the treatment of unresectable extra abdominal desmoid tumors. Elnekave et al. first reported successful results after treating four pediatric patients with unresectable, extra-abdominal desmoid tumors using drug eluting bead transarterial chemoembolization with doxorubicin over multiple treatment sessions [51]. Doxorubicin was selected as the chemotherapeutic agent of choice based on reported partial or complete response in patients treated with systemic doxorubicin [52,53]. Doxorubicin is easily loadable into drug eluting beads secondary to its ionic make up. Systemic doxorubicin administration is limited, secondary to known dose dependent cardiotoxicity. Tumor volumes were reduced by fifty four to ninety seven percent overall with a reduction of at least thirty percent volumes after the first treatment session in all cases. All patients reported symptomatic improvement following treatment. T2 signal, a marker for tumor cellularity, was eliminated on follow up magnetic resonance imaging in three out of the four patients. No serious adverse events or cardiotoxicity was observed. 

Kim et al. similarly reported positive results after treating eleven patients with extra abdominal desmoid tumors with single session drug eluting bead transarterial chemoembolization using doxorubicin [54]. The technical success rate was one hundred percent. They noted partial to near complete tumor necrosis at one month and 38.1 ± 15.3% tumor volume reduction overall. T2 signal on follow up magnetic resonance imaging was significantly reduced at one month (4.8 ± 23.6%) and overall (29.6 ± 32%) compared with baseline exams. Ten of the eleven (90.9%) patients reported improved symptom scores at a six month follow up. No serious adverse events were observed. Of note, both studies reported transient changes in the skin overlying the tumors, which was seen in all patients, and was postulated to be secondary doxorubicin pigmentation. 

## 6. Future Directions

To date, the evaluation of nearly all minimally invasive based interventions for desmoid tumors have included small, single institution cohort studies. The only prospective trial evaluating cryoablation for the treatment of extra abdominal desmoid tumors is CRYODESMO-01 [38]. Given the encouraging results of CRYODESMO-01, CRYODESMO-02 is now ongoing, prospectively comparing cryoablation versus standard medical therapy in desmoid tumor patients is progressing after the wait and see period. One challenge for any future trial in the area of desmoid tumors is the difficulty in determining a standard of care systemic therapy given the lack of data and multiple agents reported. Additional prospective, randomized, controlled, and well-powered trials are needed to evaluate the optimal therapeutic management of patients with desmoid tumors, including comparing minimally invasive procedures against or in combination with current medical, surgical, and radiation therapeutic measures. However, given the rare nature of desmoid tumors, such trials may be difficult or impossible to establish and recruit for, which may lead to institutions developing their own protocols and treatment algorithms. 

First line ablation therapy also needs further exploration, as current ablation modalities demonstrate improved tumor control when applied to smaller tumors. Additionally, in patients who are surgical candidates for tumor resection, minimally invasive ablation or embolization obviate the need for a potentially large surgical scar, which could potentially provoke new tumor formation. Furthermore, the morbidity associated with ablation and transarterial chemoembolization is typically significantly less than surgery. Finally, intra-abdominal tumors, which are usually infiltrative and located in the mesentery, are potentially highly morbid and difficult to resect surgically. Given the encouraging results of transarterial chemoembolization in extra-abdominal tumors and the precision that comes with transcatheter embolization, the treatment of intra-abdominal tumors using transarterial chemoembolization may obviate the need for a potentially dangerous surgical resection.

## 7. Conclusions

Desmoid tumors are rare, locally aggressive soft tissue tumors, without the capacity for metastasis, that have variable clinical presentation. A multidisciplinary team approach is required to provide patients with the best management of these tumors. Expectant management is appropriate for tumors that are asymptomatic or in low risk anatomic locations, with the key being the likelihood of the desmoid tumor inducing morbidity if and when it enlarges. Treatment is recommended for symptomatic patients, progressive disease, and tumors located in high risk anatomic locations. Therapeutic measures offered by interventional radiologists to treat desmoid tumors have included multiple ablation modalities and transarterial chemoembolization. While all of the aforementioned therapies have demonstrated encouraging results with similar safety profiles, cryoablation appears to be the predominant ablation modality of choice for the treatment of extra abdominal desmoid tumors. Additional prospective, randomized, controlled, and well powered trials are needed to evaluate the optimal therapeutic management of patients with desmoid tumors.

## Figures and Tables

**Table 1:** Comparison of advantages and disadvantages of ultrasound, CT, and MRI in diagnosis of desmoid tumors.

Imaging Modality	Advantages	Disadvantages
Ultrasound	No ionizing radiation Low cost Widely available Good superficial resolution	Nonspecific imaging features Limited evaluation of deep tumors
CT	Readily accessible Fast acquisition	Ionizing radiation Nonspecific imaging features
MRI	Best delineation of soft tissue planes and tumor components No ionizing radiation	High cost May not be readily accessible Nonspecific imaging features
